# Identification of potential antimicrobials against *Salmonella typhimurium* and *Listeria monocytogenes* using Quantitative Structure-Activity Relation modeling

**DOI:** 10.1371/journal.pone.0189580

**Published:** 2017-12-13

**Authors:** Ethan C. Rath, Hunter Gill, Yongsheng Bai

**Affiliations:** 1 Department of Biology, Indiana State University, Terre Haute, IN, United States of America; 2 Department of Chemistry and Physics, Indiana State University, Terre Haute, IN, United States of America; 3 The Center for Genomic Advocacy, Indiana State University, Terre Haute, IN, United States of America; Indiana University, UNITED STATES

## Abstract

The shelf-life of fresh carcasses and produce depends on the chemical and physical properties of antimicrobials currently used for treatment. For many years the gold standard of these antimicrobials has been Cetylpyridinium Chloride (CPC) a quaternary ammonium compound (QAC). CPC is very effective at removing bacterial pathogens from the surface of chicken but has not been approved for other products due to a toxic residue left behind after treatment. Currently there is also a rising trend in QAC resistant bacteria. In order to find new compounds that can combat both antimicrobial resistance and the toxic residue we have developed two Quantitative Structure-Activity Relationship (QSAR) models for *Salmonella typhimurium* and *Listeria monocytogenes*. These models have been shown to be accurate and reliable through multiple internal and external validation techniques. In processing these models we have also identified important descriptors and structures that may be key in producing a viable compound. With these models, development and testing of new compounds should be greatly simplified.

## Introduction

Foodborne illness presents a considerable risk to both public and personal health in the United States. Every year, approximately one in six United States citizens will contract some form of foodborne illness [1]. The severity and duration of the disease depends on the causative agent. According to foodsafety.gov, the majority of foodborne disease reported in the United States is caused by pathogenic bacteria. Among the many pathogenic bacterial species spread by food products, two species particularly noted for their burden on public health are *S*. *enteritidis* serovar *typhimurium* (commonly known as *S*. *typhimurium*) and *L*. *monocytogenes*.

*S*. *typhimurium* causes the gastroenteritic disease salmonellosis, which is characterized by a period of one to four days of abdominal pain, fever, and diarrhea. *S*. *typhimurium* is also capable of entering the bloodstream through the intestines and causing bacteremia. Salmonellosis accounts for a considerable amount of foodborne illness in the United States. The Centers for Disease Control and Prevention (CDC) reports that the estimated 1,027,561 annual cases of non-typhoidal salmonellosis comprise eleven percent of the total predicted annual foodborne illness cases in America. Among these annual cases, approximately 16,000 salmonellosis patients are hospitalized and 600 die [1–4]. Listeriosis, caused by *L*. *monocytogenes*, is an even more serious foodborne illness, causing bacteremia, septic abortion, and bacterial meningitis. It is predicted that 1,600 cases of listeriosis occur annually and result in 260 deaths [1, 5–10]. The prevention of foodborne diseases such as salmonellosis and listeriosis begins with the crucial step of treating food products–particularly, poultry and beef–with chemical antimicrobial agents.

Quaternary Ammonium Compounds (QACs) are a class of molecules identified by a cationic nitrogen atom. QACs that are used as antimicrobial agents often contain aliphatic, nonpolar chain tails. These compounds are widely used as general-purpose disinfectants, given their ability to impede the growth of a wide range of microorganisms [11]. The general antimicrobial mechanism for QACs is the disruption of bacterial cell walls and plasma membranes with the aliphatic tail constituents. The nonpolar chains “pinch” sections of the cell wall or membrane off; the loss of structural integrity leads to cell leakage and ultimately death of the bacterium [12–14]. There is also evidence that QACs can operate through different mechanisms, including disruption of ion channels and protein denaturation [11, 15].

Cetylpyridinium chloride (CPC) is a QAC that has shown effectiveness as an antimicrobial compound. Due to the low toxicity of CPC, it has been approved for use on chicken carcasses with the aid of propylene glycol (PEG) as a cosolvent [16, 17]. It is able to reduce aerobic colony counts significantly, and extend the shelf-life of refrigerated chicken up to 3 days compared to untreated meats[18, 19]. Although CPC has long been held to be a “gold standard” for disinfectant antimicrobial compounds, there are many issues associated with the use of CPC as a food disinfectant. Studies have shown a rising trend of resistance to certain QACs (including CPC) among bacteria[20], a negative environmental impact of CPC runoff [21], and negative health effects from ingested QAC residue left on fatty surfaces [22–26]. There is a growing need to identify new antimicrobial QACs and similar compounds that share or mimic the general antimicrobial mechanisms. These new compounds must be able to overcome immediate resistance, reduce environmental impact, and be useable on fattier surfaces without any residue.

Due to the imprecise targets of QACs and the economic advantages of computational prescreening, we turned to QSAR (Quantitative Structure-Activity Relationship) modeling to predict the antimicrobial activity of our potential compounds. QSAR models are designed through predictive tests that estimate the biological activity of untested compounds. QSAR models do this by re-expressing molecular structures as quantifiable character data; the data can then be correlated with a given bioactivity by a statistical test (i.e. MLR, PLS, Neural Networks, etc.) [27–31]. Through QSAR modeling, all of the compounds from a set can be easily compared to one another by predicted quantitative measures of biological effect [32]. This allows research groups to remove less effective compounds from consideration and readily identify the more promising compounds for further testing.

## Methods

Our methods were adapted from the procedure developed previously by the Bai lab [33].

### Finding known antimicrobials

All of the known antimicrobials for the training and test sets were acquired from three reputable and public-accessible databases: PubChem (www.pubchem.org), PubMed (www.PubMed.org), and the European Bioinformatics Institute Database (www.ebi.ac.uk). The molecules used in these sets included QACs, as well as tertiary nitrogen-based compounds with similar antimicrobial actions to QACs. The selected compounds were effective bacteriostatic agents against *S*. *typhimurium*, *L*. *monocytogenes*, or both, and held a wide range of Minimum Inhibitory Concentration (MIC) values. The Simplified Molecular Input Line Entry System (SMILES) notation and MIC value for the compounds were both reported in separate Excel files—one document for *S*. *typhimurium*, and the other for *L*. *monocytogenes*. The collected data was sorted into either the training set (85% of compounds) or the test set (15% of compounds).

### Calculating descriptors

The molecular descriptors for all data sets were calculated using the ochem.eu chemoinformatics database (https://ochem.eu). The “Calculate Descriptors” bar was accessed under the “Models” tab, and the Excel files were uploaded directly to the server. The uploaded molecules were then pre-processed; during this procedure, the salts associated with each compound structure were removed. After this stage, the molecular descriptors were selected (unless otherwise stated, the default settings for the descriptor types were not modulated). The descriptor types used were: ALogPS, GSFragment, QNPR, ISIDA fragments (fragment length was set as 2 to 10), and E-state (all boxes checked apart from “Extended indices—experimental”). These were selected from the entire set as they did not contain any 3D descriptors. This selection was made to avoid errors that occur with 3D descriptors calculation. Any compounds still experiencing errors in calculation were deleted from the descriptor sets, and the remaining sets were saved as.csv files. Resulting in 1356 descriptors for each compound.

### Descriptor output modification and data normalization

The.csv files for *S*. *typhimurium* and *L*. *monocytogenes* were modified before QSAR use. Column headings with no empirical data (i.e. Comments) were deleted from the files and a new column containing the log of each compound’s MIC (logMIC) was inserted into the documents. Finally, each compound was randomly assigned to either the training or test groups for both pathogens. 85% of the total molecules in the data sets were used as the training group and the remaining 15% were used in the test group. In all, the *S*. *typhimurium* file contained 26 compounds in the training group and 6 compounds in the prediction group; the *L*. *monocytogenes* file contained 37 compounds in the training group and 7 compounds in the prediction group. The compounds were assigned numbers in a new “split” column by their status; “1” indicated assignment to the training group, “2” indicated molecules from the testing group. These modified.csv files were then uploaded to the normalizeTheData (v.1.0) data normalization tool [34] (http://teqip.jdvu.ac.in/QSAR_Tools/#ADInHouse), which produced a new version of the.csv files with adjusted molecular descriptor values. Last, the.csv files were reformatted as.txt files for compatibility with the QSAR program.

### QSAR modeling

After the file modifications, the.txt data files for *S*. *typhimurium* and *L*. *monocytogenes* were imported separately into the QSARINS program [35, 36](qsar.it). All compounds in the test set were manually deleted from the uploaded file. Following this, the training compounds were run through the software’s internal filters. The internal filters were used to remove descriptors that had under 80% consistency throughout the data set and those that were 95% correlated. The internal filters removed the majority of the descriptors for all QSAR models. The next step involved a setup procedure for the QSARINS equation in which the remaining molecular descriptors were selected as the variables and the log(MIC) was selected as the response. The in-program Genetic Algorithm was then applied to select the top models for each iteration of descriptors based on their Q^2^ (average leave-one-out fit) values. The number of iterations (number of times the Genetic Algorithm is run) is equal to 1/5th the total number of compounds in the test set (as recommended by Eriksson *et al*. [37]); the number of combinations (of molecular descriptor variables) is equal to the number of molecular descriptors that remain after the preliminary internal filtering step. After the Genetic Algorithm is applied QSARINS displays the top 5 models from each iteration. The algorithm was set to run for 20 iterations with 500 generations processed for each iteration.

### Internal selection

These models were then sorted according to their respective R^2^ and R^2^-Q^2^ values. The default model parameters for *S*. *typhimurium* and *L*. *monocytogenes* were the same for both; however, as QSARINS produced models with different value ranges for each, the parameters were slightly modulated to reduce the overall number of models that were considered. Our cutoff values were R^2^ ≥ 0.75 and R^2^-Q^2^ ≤ 0.10. These numbers were elevated from the less conservative cutoffs presented by most other researchers [27, 28, 31, 37]. Therefore, only the top models selected by internal statistics were used for further analysis.

### External validation

The saved model file for the selected top models were retrieved from the “models” bar and loaded onto QSARINS. The number of rows was adjusted to reflect the number of compounds within the test set. The compounds and relevant molecular descriptor data were then loaded into the model. The model predicted the log(MIC) of the test compounds; these predicted log(MIC) values were contrasted against the experimental log(MIC) values by percent error analysis and the R^2^ (linear trend fit) of the predicted compounds.

### Predictions

Once the final top models were selected and combined as described above, each model was then used on the prediction set collected from the Bai lab [33]. A set of 835 compounds was collected from PubChem based off the top results from a similar identity search using CPC as the query. These compounds were then filtered based on the model applicability domain for each model, any model outside the applicability domain was removed from the final results. The consensus models for each bacterium were also applied to this data set. Any compound within the applicability domain for 75% of the combined models were kept, all other compounds were removed from the final set.

### Consensus modeling

The consensus models were built using the average of all predicted values for the models that met the following criteria. First, the single worst model of each set was removed (77 for *S*. *typhimurium* and 84 for *L*. *monocytogenes*). Models were then included for the selected consensus if they met the following criteria, a cutoff of external validation R^2^ ≥ 0.60 and percent error ≤ 10% for *S*. *typhimurium* and external validation R^2^ ≥ 0.90 and percent error ≤ 35% for *L*. *monocytogenes*. This technique was also used to combine the predictions from the final model identified for each bacterial species.

### Structural similarity comparison

In order to better understand the relationship between the top compounds identified by the QSAR models developed for this project and for our previous study of *E*. *coli*, we looked at similarity and substructures shared between the top 50 compounds from each predicted set using SIMCOMP2 [38–40]. In order to use this tool, all SMILES structures were converted into.mol files using molview [41]. The.mol files for each prediction set were then concatenated to produce a single.mol file with all 50 structures. Each set was then analyzed pairwise against the other two sets using SIMCOMP2 with the following parameters: global search, bond based docking matched by KEGG atom, post-processing for all SCCSs matched by atom, and a cutoff value of 0.60. This data was further filtered to cutoff score of 0.75 and limited to structures that matched with structures across all data sets [38–40].

## Results

### S. typhimurium models

**Fig 1:**
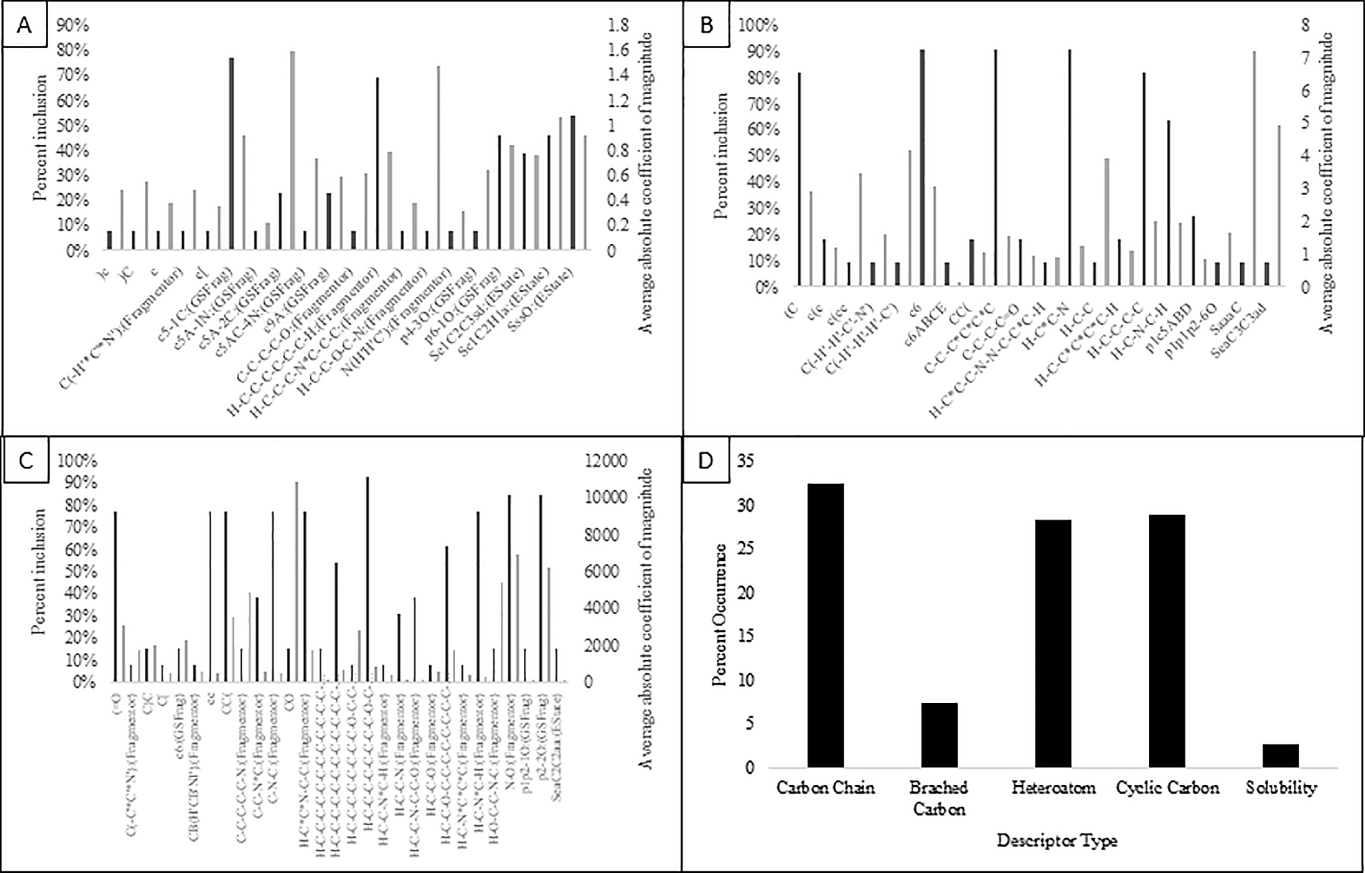
Descriptor distribution for top QSAR models. Distribution of chemical descriptors (black) and their respective average absolute coefficient magnitudes (black), in top models for *S*. *typhimurium* (A) and *L*. *monocytogenes* (B). Descriptor distribution normalized to the total number of models observed for each bacteria. (C) Descriptor distribution (black) and average absolute coefficient magnitudes (grey) based on previously created models against *E*. *coli*.[33] Most descriptors are in canonical SMILES, using single letters to represent atoms in a molecule, “*” denote any atom, “‘“ represent potential atoms, “()” represent branches in a molecule, numbers represent joining points in ring structures, “=“ represent double bonds, and lower case letters are atoms involved in aromatic strucutres. (D) Distribution of descriptor types across all top models. Descriptors that were pertinent to multiple bins were included in all potential bins.

Detection of potentially new food safe antimicrobials first starts with looking at the best current technologies being applied. We performed a literature search to find all available data on QACs and QAC-like compounds that have been tested against *S*. *typhimurium*. Specifically we looked for studies pertaining to minimum inhibitory concentrations (MIC) of compounds against this bacterium. MIC is a measure of the effectiveness of an antimicrobial by the lowest concentration that inhibits growth, making the most effective compounds to have the lowest MIC. Although previous individual studies provide a more stable and accurate model building sets, no single study had a wide enough range of MIC values or a wide enough range of different molecules. Therefore, we decided to utilize a group of 32 compounds that were collected from 8 different sources [42–48]. The compounds we chose had at least a single nitrogen with 3 or more constituent groups, beyond that these compounds had variable numbers of carbon, benzene rings, oxygen, and other distinct structural differences. Their MIC values ranged from 3 μg/ml to 1071.1 μg/ml. Having a wide range of MIC values and a wide range of structural differences increases our confidence in our results. These structures were used to develop over 300 models using the QSARINS software. This software produces predictive models using a multiple linear regression (MLR) based approach paired with a genetic algorithm for descriptor selection. The algorithm iterates through models based off a number of descriptors based on the iteration number. It then substitutes descriptors out from each individual model based on the given mutation rate. The top 10 models, based on the average linear fit loss of one (Q^2^) for each model, are kept for the next round of mutations. Each iteration cycles through 500 generations. After each iteration a list of the top 5 models of each iteration is kept for further analysis. 20 iterations were done for each model. The top models, as determined by internal selections (Table 1), were applied to external datasets to produce predicted MIC values. The best of these models were selected via filtering using their R^2^ (linear trend fit) and R^2^-Q^2^ values (difference between linear trend fit and average leave-one-out fit). Within these models the most frequent descriptors that were shared between all of these top compounds included a cyclic SP2 hybridized 5 carbon ring attached to a methyl group and a minimum 6 carbon chain (Fig 1A). The incidence of use of these descriptors do not seem to be correlated to the magnitudes of the coefficients however. Our data shows that the descriptors that have the highest coefficient values are cyclic carbons attacked to a Nitrogen atom and a carbon chain containing oxygen attached to a Nitrogen. These descriptors only appear in ~25% and ~10%, respectively, of the top models. As such, a singular descriptor cannot be ascertained as being most important in predicting MIC values of potential QACs.

**Table 1:** Internal and external validation data for top S. typhimurium and L. monocytogenes Models.

	Internal Statistics			External Validations	
Model Number	R^2^	Q^2^	R^2^-Q^2^	R^2^	Average Percent Error
*S*. *typhimurium*					
77	0.86	0.79	0.07	0.06	15.49
79	0.86	0.80	0.06	0.66	9.23
78	0.86	0.79	0.06	0.62	9.40
80	0.85	0.80	0.05	0.36	14.32
76	0.85	0.79	0.07	0.70	7.42
75	0.85	0.79	0.07	0.63	9.96
74	0.85	0.78	0.07	0.65	9.00
73	0.85	0.78	0.07	0.66	7.63
71	0.85	0.78	0.07	0.65	10.47
72	0.85	0.78	0.07	0.15	9.53
68	0.85	0.77	0.07	0.51	10.18
70	0.85	0.79	0.05	0.40	10.43
67	0.84	0.77	0.07	0.53	10.58
Consensus	N/A	N/A	N/A	0.53	10.58
Consensus (selected)	N/A	N/A	N/A	0.62	8.73
Consensus (worst deleted)	N/A	N/A	N/A	0.60	8.38
*L*. *Monocytogenes*					
89	0.91	0.84	0.07	0.93	33.08
92	0.91	0.85	0.06	0.84	48.87
90	0.90	0.84	0.07	0.95	33.38
87	0.90	0.84	0.07	0.82	52.44
91	0.90	0.84	0.06	0.80	51.75
84	0.90	0.83	0.07	0.43	42.08
88	0.90	0.84	0.06	0.84	48.56
85	0.90	0.83	0.07	0.79	50.05
86	0.90	0.83	0.07	0.77	43.77
83	0.89	0.82	0.07	0.88	46.32
82	0.85	0.75	0.10	0.89	54.19
Consensus	N/A	N/A	N/A	0.88	41.51
Consensus (selected)	N/A	N/A	N/A	0.94	32.73
Consensus (worst deleted)	N/A	N/A	N/A	0.90	41.78

**Fig 2:**
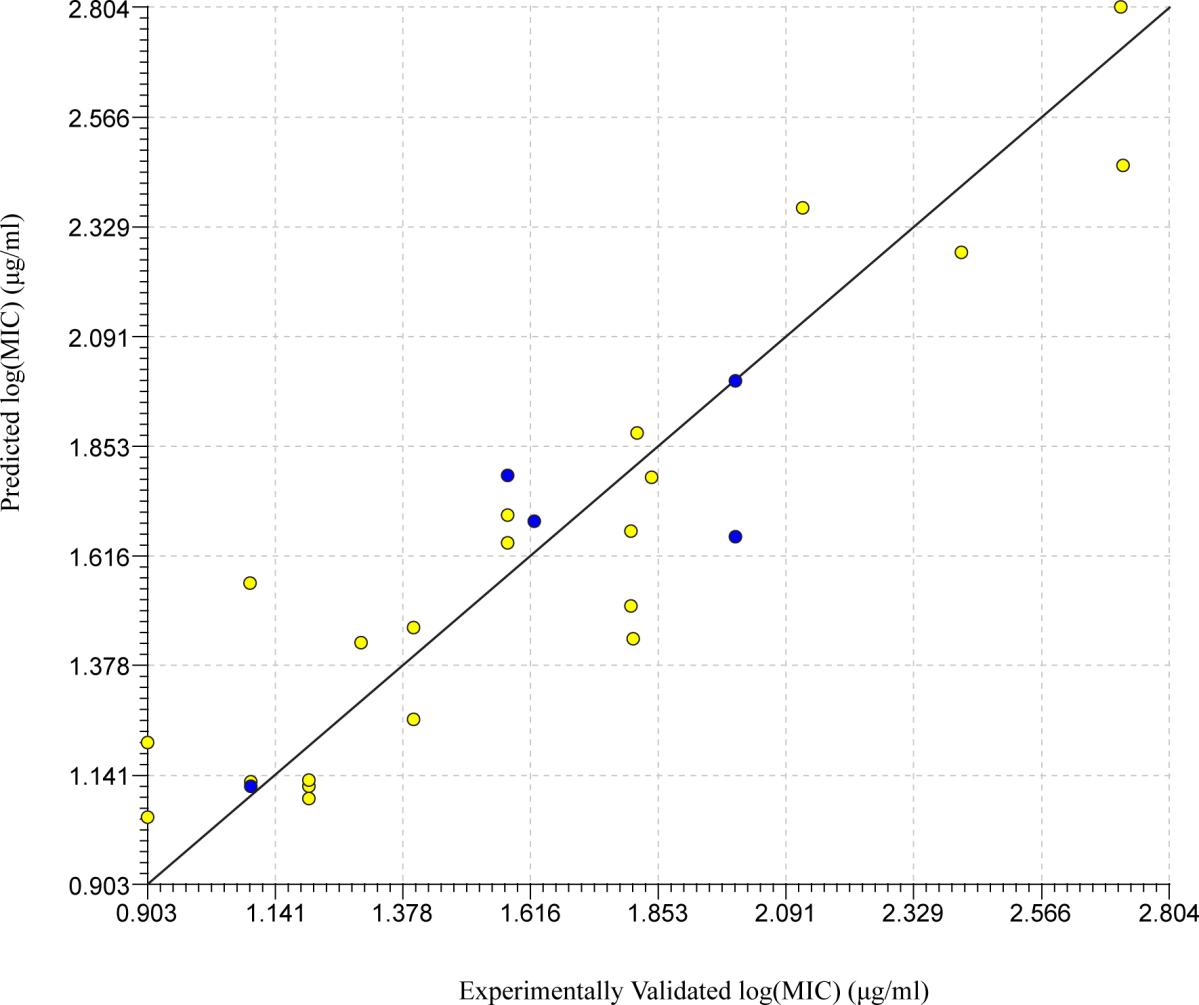
Linear regression for top model against *S*. *typhimurium*. Regression of experimentally validated and model predicted log(MIC) values for the top QSAR model (model 76) for *S*. *typhimurium* selected through internal and external validations. The yellow dots represent the training set while the blue dots represent the test set.

Using external validations and our own internal filters, a final list of 14 models was collected for *S*. *typhimurium* (Table 1). A number of these models were then used to develop three separate consensus models (Table 1). These consensus models were developed in hope of creating a more accurate model. After looking over the data, we determined that model 76 was the best possible model having the highest R^2^ (by 0.04) and lowest percent error (by 0.21%) of the external validation set. Its internal statistics were not the best of the set, however they were comparable to the rest of the models. The regression of this model can be seen in Fig 2. Of all the potential models, 76 really stood out as the most potentially accurate and consistent model. It is interesting to note that the R^2^ values of these models diminished across the validation sets. This could be due to the selection of test compounds being on the high end of the applicability domain, unlike the *L*. *monocytogenes* set. Having determined the optimal model, we began producing predictions using the QSARINS program.

### S. typhimurium predictions

Predictions of the QSAR model that we selected were run on the dataset previously curated by Rath *et al*. based on a sub structure search of CPC [33]. This list contains no samples that have a previously predicted or experimentally validated MIC, unlike the test set used for external validations. Out of the 834 compounds, we identified the top 10 compounds that fit within the applicability domain of model 76 (Table 2). These compounds are similar in that they all contain one or more ring structures and some form of nitrogen. It’s interesting to note that there is not a positively charged nitrogen in each molecule.

**Table 2:** Top 10 compounds against S. typhimurium in terms of log(MIC).

SMILES Structure of Potential Compounds	log(MIC) (μg/ml)	SMILES Structure of Potential Compounds	log(MIC) (μg/ml)
*S*. *typhimurium* (model 76)			
C[C@@]12CC[C@]3(CC[C@H](C3C1CCC4[C@]2(CCC5[C@@]4(CC[C@@H](C5(C)C)O)C)C)C (= C)C[N+]6 = CC = CC = C6)CO	0.606	C[C@@]12CC[C@]3(CC[C@H](C3C1CCC4[C@]2(CCC5[C@@]4(CC[C@@H](C5(C)C)O)C)C)C (= C)C[N+]6 = CC = CC = C6)CO	0.606
CC1 = CC (= C[N+] (= C1)CC (= C)[C@@H]2CC[C@]3(C2C4CCC5[C@]6(CC[C@@H](C(C6CC[C@]5([C@@]4(CC3)C)C)(C)C)O)C)CO)C	0.6082	CC1 = CC (= C[N+] (= C1)CC (= C)[C@@H]2CC[C@]3(C2C4CCC5[C@]6(CC[C@@H](C(C6CC[C@]5([C@@]4(CC3)C)C)(C)C)O)C)CO)C	0.6082
C[C@@]12CC[C@]3(CC[C@H](C3C1CCC4[C@]2(CCC5[C@@]4(CC[C@@H](C5(C)C)O)C)C)C (= C)C[N+]6 = CC = C(C = C6)N(C)C)CO	0.6207	C[C@@]12CC[C@]3(CC[C@H](C3C1CCC4[C@]2(CCC5[C@@]4(CC[C@@H](C5(C)C)O)C)C)C (= C)C[N+]6 = CC = C(C = C6)N(C)C)CO	0.6207
C[C@@]12CC[C@]3(CC[C@H](C3C1CCC4[C@]2(CCC5[C@@]4(CC[C@@H](C5(C)C)O)C)C)C (= C)C[N+]6 = CC = CC (= C6)CO)CO	0.6251	C[C@@]12CC[C@]3(CC[C@H](C3C1CCC4[C@]2(CCC5[C@@]4(CC[C@@H](C5(C)C)O)C)C)C (= C)C[N+]6 = CC = CC (= C6)CO)CO	0.6251
C[C@@]12CC[C@]3(CC[C@H](C3C1CCC4[C@]2(CCC5[C@@]4(CC[C@@H](C5(C)C)O)C)C)C (= C)C[N+]6 = CC = C(C = C6)CO)CO	0.6273	C[C@@]12CC[C@]3(CC[C@H](C3C1CCC4[C@]2(CCC5[C@@]4(CC[C@@H](C5(C)C)O)C)C)C (= C)C[N+]6 = CC = C(C = C6)CO)CO	0.6273

### L. monocytogenes models

Much like *S*. *typhimurium*, a single study with enough antimicrobial information was not found for *L*. *monocytogenes*. The final list of compounds was taken from 12 sources and contained 49 unique compounds [49–57]. These compounds were similar in structural depth to the previous set (above) of compounds but ranged in MIC from 0.0005 μg/ml to 50 μg/ml. This resulted in an even larger list of potential models than the list for *S*. *typhimurium*. In the top 11 models there were four descriptors were involved in > 90% of the top models. These include a constituent group that starts with a single carbon, a 6 member carbon ring, a 6 carbon chain, and two carbons leading to a nitrogen (Fig 1B). The two descriptors that showed the greatest coefficients were the two descriptors containing Sulfur. These two descriptors were only involved in ~10% of the top compounds. Through this data we cannot ascertain a singular descriptor that is most important for MIC prediction across the top models.

**Fig 3:**
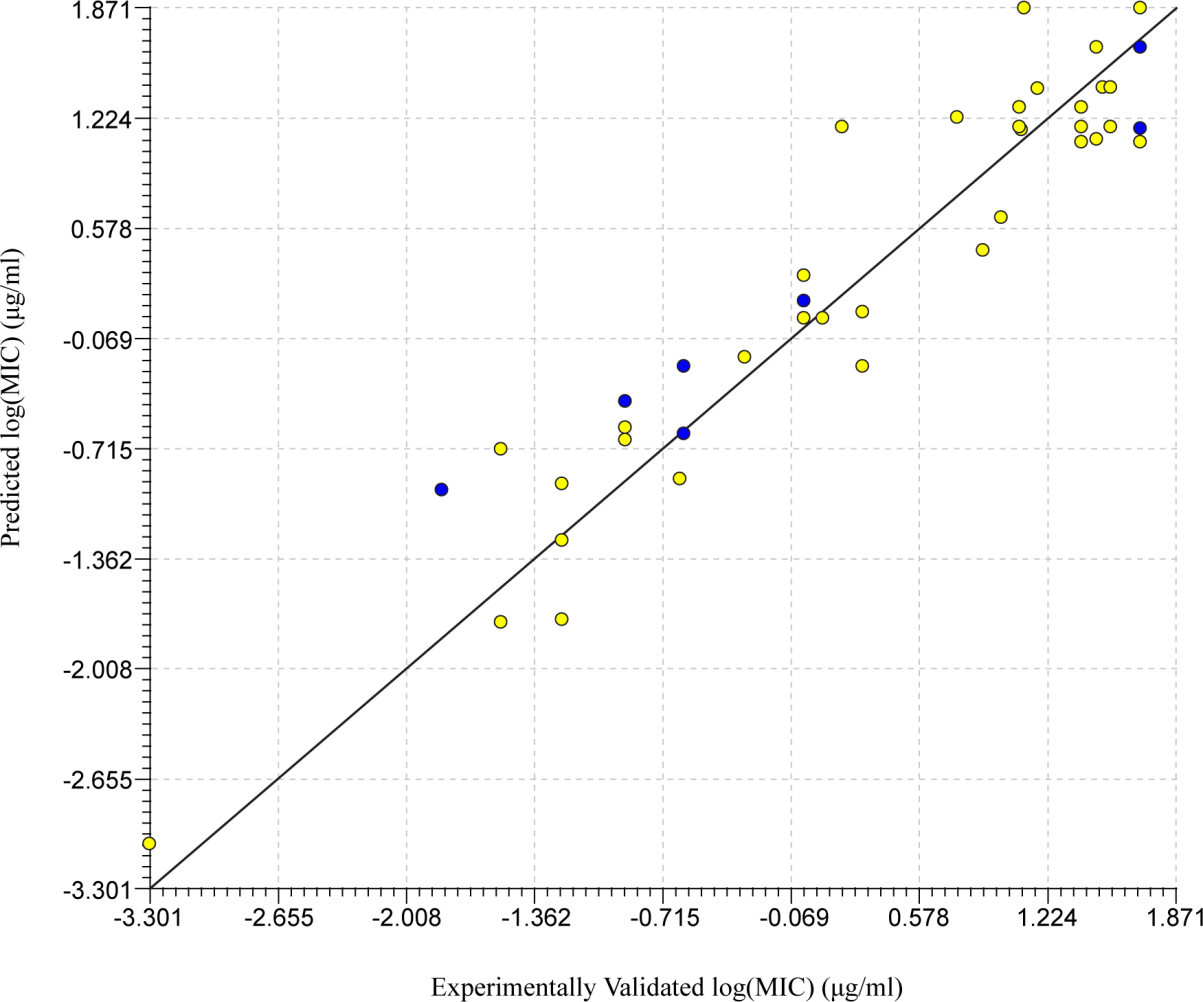
Linear regression for top model against *L*. *monocytogenes*. Regression of experimentally validated and model predicted log(MIC) values for the top QSAR model (model 90) for *L*. *monocytogenes* selected through internal and external validations. The yellow dots represent the training set while the blue dots represent the test set.

Using the same internal filters and an external validation set the final list of 11 compounds was curated (Table 1). These models were used to create three additional consensus models to be considered for the final prediction. These models have a much higher margin of error than that of the *S*. *typhimurium* models, this could be due to a greater variance in the structures of the compounds used in the training and test sets. Model 90 was selected as the optimal model of this set. It was the top single model for external validation R^2^ and the second lowest percent error by only 0.30% (Fig 3). Although the selected consensus model had reduced error and a very close R^2^, we chose a singular model for ease and efficiency in prediction.

### L. monocytogenes predictions

We ran the predictions of the same 835 compounds through the selected model in order to determine the best possible compounds from this set. The top ten compounds that lay within the applicability domain of model 90 are shown in Table 3. These compounds have many structural similarities to those detected by the *S*. *typhimurium* based model.

**Table 3:** Top 10 against L. monocytogenes in terms of log(MIC).

SMILES Structure of Potential Compounds	log(MIC) (μg/ml)	SMILES Structure of Potential Compounds	log(MIC) (μg/ml)
*L*. *monocytogenes* (model 90)			
CCCCCCCCCCCCCCCC[N+]1 = CC = C(C2 = CC = CC = C21)CC(C3 = C(NC4 = CC = CC = C43)C)O	-1.8558	CCCCCCCCCCCCCCCC[N+]1 = CC = C(C2 = CC = CC = C21)CC(C3 = C(NC4 = CC = CC = C43)C)O	-1.8558
CC1 = CC (= C[N+] (= C1)CC (= O)C2 = CC3 = C(C = C2)C4 = CC = CC = C4C3)C.[Br-]	-1.7315	CC1 = CC (= C[N+] (= C1)CC (= O)C2 = CC3 = C(C = C2)C4 = CC = CC = C4C3)C.[Br-]	-1.7315
CC1 = CC = CC2 = NC (= C(N12)NC(C)(C)CC(C)(C)C)C3 = CC4 = CC = CC = C4C5 = CC = CC = C53	-1.7252	CC1 = CC = CC2 = NC (= C(N12)NC(C)(C)CC(C)(C)C)C3 = CC4 = CC = CC = C4C5 = CC = CC = C53	-1.7252
CC1 = C[N+] (= CC = C1)CCCCCC2 = CC (= CC = C2)CCCCC[N+]3 = CC = CC (= C3)C	-1.7101	CC1 = C[N+] (= CC = C1)CCCCCC2 = CC (= CC = C2)CCCCC[N+]3 = CC = CC (= C3)C	-1.7101
CC1 = C[N+] (= CC = C1)CCCCCC2 = CC (= CC = C2)CCCCC[N+]3 = CC = CC (= C3)C.[Br-].[Br-]	-1.7101	CC1 = C[N+] (= CC = C1)CCCCCC2 = CC (= CC = C2)CCCCC[N+]3 = CC = CC (= C3)C.[Br-].[Br-]	-1.7101

### Combining all predictions

Having three separate QSAR models (the two detailed here and one from a previous study for *E*. *coli* [33]) for each individual target species is effective in providing highly accurate and reliable predictions. Alone these models are unable to produce a direct consensus on a singular set of potential compounds, and cannot be directly combined without making incomplete assumptions of the effectiveness of known compounds. In order to gain a better understanding of the predicted compounds’ effectiveness across a range of pathogenic bacteria. This information was gathered through two separate approaches. The first approach focuses on using the average predicted MIC across all models and the later utilizes similarity scoring to find general structural similarities of top compounds from each model.

One approach to combining the predictions of the models is to follow our consensus approach. By averaging the log(MIC) for each compound across all three predictions we are able to draw conclusions as to the general effectivity of the compounds. The top 5 compounds are reported in Table 4. These compounds have a structure similar to CPC with an aromatic head and a long hydrophobic tail.

**Table 4:** Top 5 compounds according to average log(MIC) from all 3 predictive models.

SMILES Structure	*E*. *coli*	*S*. *typhimurium*	*L*. *monocytogenes*	Average log(MIC)
CCCCCCCCCCCC(CCCC[N+]1 = CC = CC = C1)C (= O)N	1.04	1.39	-1.00	0.48
CCCCCCCCCCCCCCC(C)[N+]1 = CC = CC = C1	1.13	1.37	-1.02	0.50
CCCCCCCCCCCCCCC(C (= O)[O-])[N+]1 = CC = CC = C1	1.16	1.45	-1.04	0.52
CCCCCCCCCCCCCCCC (= O)[N+]1 = CC = C(C = C1)N(C)C	0.97	1.44	-0.71	0.57
CCCCCCCCCCCCCCCC[N+]1 = CC = C(C = C1)C (= O)N	1.02	1.41	-0.68	0.58

**Fig 4:**
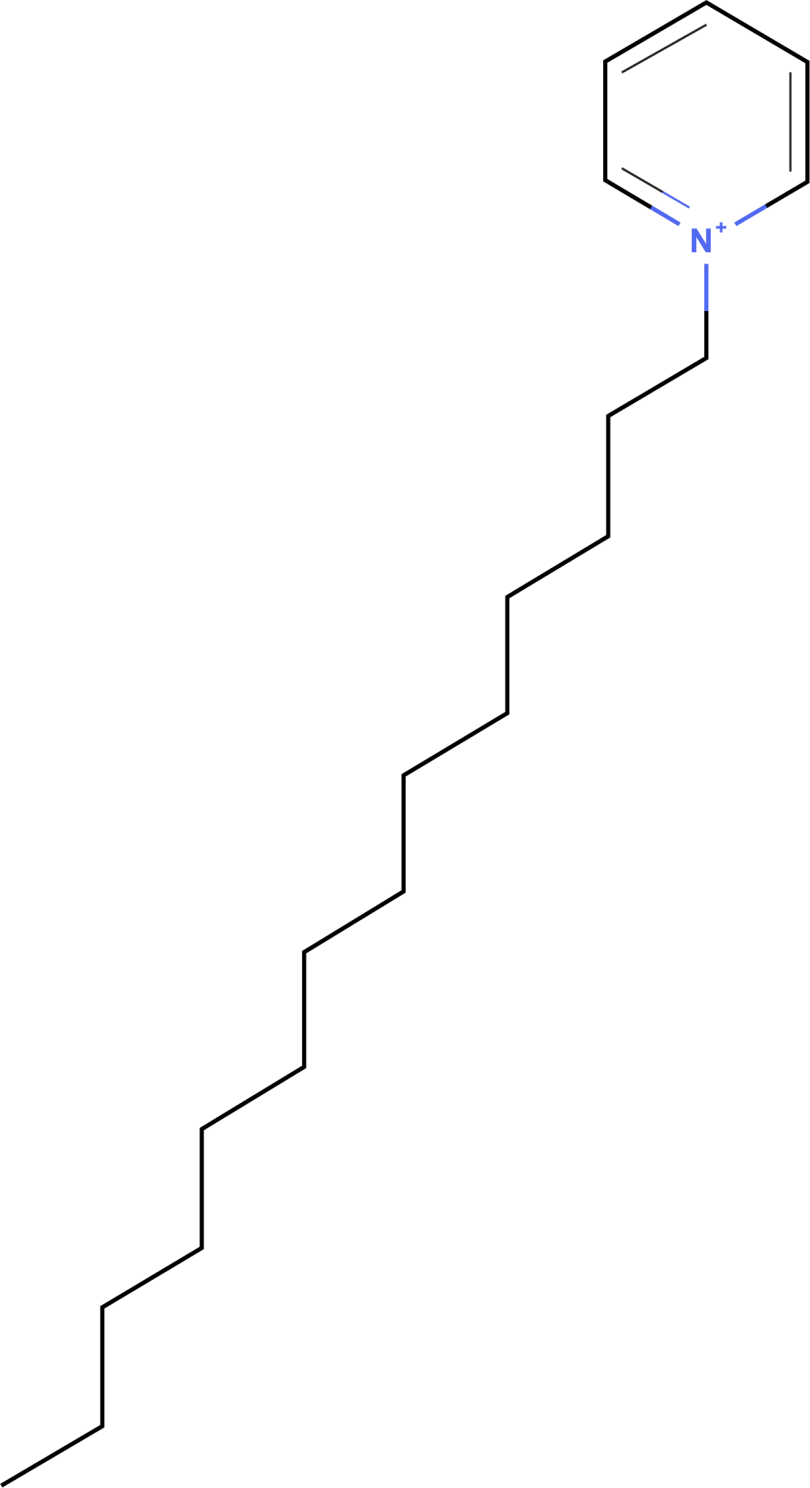
Substructure amongst top compounds predicted by QSAR modelling. Major substructure detected by SIMCOMP2 software for the top similar structures from their prediction sets.

When trying to define possible compounds that can effect multiple species of pathogenic bacteria, it is important to look at the structural similarities between the top compounds from each predictive model. In order to find the regions of similarity and the degree of similarity between each set, we turned to a pairwise comparison through SIMCOMP2, an online tool provided by KEGG. In order to expand the possibilities of similar compounds, we expanded our potential list to the top 50 compounds predicted for each species. Multiple similar structural components were identified within pairwise comparisons, however only one major structure was identified for the compounds with at least 0.75 similarity between all three predictions (Fig 4). A search of the most similar compounds returned 1 *L*. *monocytogenes* compound, 3 *S*. *typhimurium* compounds, and 9 *E*. *coli* compounds (Table 5). The actual structures of these compounds can be found in S1 Table. These compounds provide a good launching point for experimental validation, or rational design of new compounds.

**Table 5:** Pairwise similarity comparisons between top 50 predicted compounds for three bacterial species.

Listeria Compound	Similarity Score (*L*. *monocytogenes* & *S*. *typhimurium)*	*S*. *Typhimurium* Compound	Similarity Score (*S*. *typhimurium* & *E*. *coli*)	*E*. *coli* compound	Similarity Score (*E*. *coli* & *L*. *monocytogenes)*
35	0.77	37	0.78	16	0.84
35	0.77	37	0.78	23	0.84
35	0.77	37	0.78	24	0.84
35	0.77	37	0.78	35	0.84
35	0.77	37	0.78	37	0.84
35	0.77	37	0.78	39	0.84
35	0.77	39	0.78	16	0.84
35	0.77	39	0.78	23	0.84
35	0.77	39	0.78	24	0.84
35	0.77	39	0.78	35	0.84
35	0.77	39	0.78	37	0.84
35	0.77	39	0.78	39	0.84
35	0.77	37	0.76	36	0.80
35	0.77	37	0.76	38	0.80
35	0.77	37	0.76	40	0.80
35	0.77	39	0.76	36	0.80
35	0.77	39	0.76	38	0.80
35	0.77	39	0.76	40	0.80
35	0.75	36	0.78	16	0.81
35	0.75	36	0.78	23	0.81
35	0.75	36	0.78	24	0.81
35	0.75	36	0.78	35	0.81
35	0.75	36	0.78	37	0.81
35	0.75	36	0.78	39	0.81
35	0.75	36	0.78	37	0.75
35	0.75	36	0.76	36	0.77
35	0.75	36	0.76	38	0.77
35	0.75	36	0.76	40	0.77

## Discussion

The development of an accurate and reliable computation tool for the development and identification of potential food safe antimicrobials is paramount for increasing the efficiency of this process, especially for those under the QAC and QAC-like umbrella. Using currently available QAC antimicrobial data we developed two models that focus of the effectiveness of these compounds on *S*. *typhimurium* and *L*. *monocytogenes* respectively. These models were produced using an optimized descriptor set and the built in genetic algorithm (GA) within the QSARINS software. The models we have identified in this study have shown both accuracy (percent error) and reliability (Q^2^) against the test and training sets. We have confidence these are the optimal models for the datasets that are currently available.

We have identified the top used descriptors for each data set, potentially providing great insight into the important structural components of effective QACs. From the data collected, long chains of carbon (at least 5 carbons) followed by both cyclic rings and the inclusion of heteroatoms were shown to be the most frequent descriptors for top models (Fig 1D) Consequently, we can assume that the carbon structure of these compounds is very important to their antimicrobial effects. Due to the mechanism that QACs rely on, this relationship not only makes sense, but adds to the confidence that we have in the accuracy of our models.

These models and the model produced for a previous publication have uncovered a number of top rated compounds that have potential as food safe antimicrobials [33]. There were no compounds that were shared amongst the top ten compounds for each organism; however, there were many structural similarities observed (Tables 2 and 3). Each top ten set contained at least one straight chain compound with a hexane ring in the head region, this is similar to most commonly used QACs. These compounds may be a good immediate replacement for CPC in meat processing but may not provide much difference in terms of solubility, residue, and in overcoming potential antimicrobial resistance.

There are other compounds in these sets that do show potential for overcoming these issues. Within these sets there are a group of compounds that have a larger “head”, usually composed of two fused rings with one to three nitrogens (not usually charged) and a large set of fused carbon rings as the “tail”. This tail is slightly reminiscent of the structure of most cholesterols and other steroids. This structure gives us confidence that these compounds might be able to interact with bacterial membranes in a similar manner to their straight chained cousins. If this is the case, it is possible that these compounds could easily overcome QAC bacterial resistance and could possibly reduce the issues of residue. Unfortunately, these compounds would be much harder to dissolve in water as they have very large hydrophobic regions. Regardless, these compounds have potential in replacing the current QACs being used for food safe decontamination as they have a chance to overcome bacterial resistance.

Through the use of both a consensus between the different bacterial models and through structural similarity detected between the top 50 predicted compounds from each model we were able to determine the most important structural elements to help narrow the list of potential compounds. All compounds have a structure that is nearly identical to the structure of CPC, this shows a high degree of confidence in our ability to detect top compounds using either method. Unfortunately, these compounds do not give us any structures that will be greatly effective in reversing and overcoming antimicrobial resistance. We attempted to further limit this list by looking at the toxicity of each compound, the more toxic compounds being less likely to be good food safe antimicrobials. None of the compounds in Table 4 or Table 5 had any available toxicity data and all predictions of toxicity from open-source prediction tools were too varied to draw any adequate conclusions. Future experimental validation for our computationally identified compounds would provide a final list of candidate food antimicrobials.

While our current predictions are based on a wide ranging list, these models could be reapplied to a more targeted list of potential compounds. This would provide an extra filtering step to any study that wishes to experimentally test any potential QAC/QAC-like compounds. Furthermore, our top potential compounds could also be tested in a similar manner.

Our study focuses on a more accurate model for less different structures. It is of our belief that these structures will still be able to overcome some of these issues even immediate resistance (as their binding affinity to e-flux pumps may be reduced), although this approach would not be able to overcome long term resistance. In terms of lipophilicity, some of our predicted compounds contain more hetero-atoms which lead to increased polarity which would affect their ability to be removed from fattier surfaces using water based washes/rinses. Environment impact of these compounds cannot be currently ascertained however.

The discovery and development of new QAC/QAC-like compounds is vital in the preservation of food and in the management of pathogenic microbes on the surface of foods. Without more research into potential compounds, antibiotic resistance and other current problems will continue to be a detriment to the food industry and in turn the consumer. Any new compound that can overcome the issues of current food safe antimicrobials will become a gold standard for all other antimicrobials. Although our current list is not perfect for replacement of CPC, our model can be applied to other QAC and QAC-like compounds to increase the viability of future studies and reduce the cost of bulk sampling of these lists.

## Supporting information

S1 Table Average top structures detected by combining all top bacterial models. (DOCX)

## References

[1] Scallan E, Hoekstra RM, Widdowson MA, Hall AJ, Griffin PM. Foodborne Illness Acquired in the United States Response. Emerg Infect Dis. 2011;17(7):1339–40. 10.3201/eid1701.P11101 PMC3375761 21192848

[2] CDC. Braenderup infections linked to nut butter: Clinical Features/ Signs and Symptoms. In: Services USDoHaH, editor. Atlanta, Georgia: CDC; 2014.

[3] CDC. Foodborne Diseases Active Surveillance Network (FoodNet): FoodNet Surveillance Report for 2012 In: Services USDoHaH, editor. Atlanta, Georgia: CDC; 2014.

[4] CDC. Suspecting Foodborne Illnesses in Special Populations: Quick Facts for Providers. In: Services USDoHaH, editor. Atlanta, Georgia: CDC; 2012.

[5] Schlech WF 3rd, Lavigne PM, Bortolussi RA, Allen AC, Haldane EV, Wort AJ, et al Epidemic listeriosis—evidence for transmission by food. N Engl J Med. 1983;308(4):203–6. doi: 10.1056/NEJM198301273080407 6401354 10.1056/NEJM198301273080407

[6] Dalton CB, Austin CC, Sobel J, Hayes PS, Bibb WF, Graves LM, et al An outbreak of gastroenteritis and fever due to Listeria monocytogenes in milk. New Engl J Med. 1997;336(2):100–5. doi: 10.1056/NEJM199701093360204 8988887 10.1056/NEJM199701093360204

[7] Jackson KA, Iwamoto M, Swerdlow D. Pregnancy-associated listeriosis. Epidemiol Infect. 2010;138(10):1503–9. doi: 10.1017/S0950268810000294 20158931 10.1017/S0950268810000294

[8] Riedo FX, Pinner RW, Tosca MD, Cartter ML, Graves LM, Reeves MW, et al A Point-Source Foodborne Listeriosis Outbreak—Documented Incubation Period and Possible Mild Illness. J Infect Dis. 1994;170(3):693–6. 8077731 10.1093/infdis/170.3.693

[9] Ryser ET, Marth EH. Listeria, Listeriosis, and Food Safety, Third Edition 3 ed. Boca Raton, Florida: Taylor and Francis Group.

[10] Silk BJ, Date KA, Jackson KA, Pouillot R, Holt KG, Graves LM, et al Invasive listeriosis in the Foodborne Diseases Active Surveillance Network (FoodNet), 2004–2009: further targeted prevention needed for higher-risk groups. Clin Infect Dis. 2012;54 Suppl 5:S396–404. 22572660 10.1093/cid/cis268

[11] Liu J, Ling JQ, Wu CD. Cetylpyridinium chloride suppresses gene expression associated with halitosis. Arch Oral Biol. 2013;58(11):1686–91. doi: 10.1016/j.archoralbio.2013.08.014 24112735 10.1016/j.archoralbio.2013.08.014

[12] Ioannou C, Hanlon G, Deyner S. Action of Disinfectant Quaternary Ammonium Compounds against Staphylococcus aureus. Antimicrobial Agents and Chemotherapy. 2007;51:296–306. doi: 10.1128/AAC.00375-06 17060529 10.1128/AAC.00375-06 PMC1797692

[13] Wessels S, Ingmer H. Modes of action of three disinfectant active substances: A review. Regulatory Toxicology and Pharmacology. 2013;67:456–67. doi: 10.1016/j.yrtph.2013.09.006 24080225 10.1016/j.yrtph.2013.09.006

[14] Deyner S, Maillard J. Cellular impermeability and uptake of biocides and antibiotics in Gram-negative bacteria. Journal of Applied Microbiology, Symposium suppliment. 2002;92:35S–45S. 12000611

[15] Taheri-Kafrani A, Rastegari AA, Bordbar AK. The unfolding process of apo-human serum transferrin in the presence of cetylpyridinium chloride: an isothermal titration calorimetry study. Acta Chim Slov. 2014;61(3):645–9. 25286222

[16] Gilbert P, Moore LE. Cationic antiseptics: diversity of action under a common epithet. J Appl Microbiol. 2005;99(4):703–15. doi: 10.1111/j.1365-2672.2005.02664.x 16162221 10.1111/j.1365-2672.2005.02664.x

[17] Kwak KY, Nakata Y, inventorsMethod for decreasing absorption of microbicides on fiber assemblies by surfactants. Japan1999.

[18] Bai Y, Coleman K, Waldroup A. Effect of Cetylpyridinium Chloride (Cecure CPC Antimicrobial) on the Refrigerated Shelf Life of Fresh Boneless, Skinless Broiler Thigh Meat. International Journal of Poultry Science. 2007;6:91–4.

[19] Gilbert C, Bai Y, Jiang H. Microbial Evaluation of Cecure-Treated (Post-Chill) Raw Poultry Carcasses and Cut-up Parts in Four Commercial Broiler Processing Facilities. International Journal of Poultry Science. 2015;14:120–6.

[20] Buffett-Bataillon S, Tattevin O, Bonnaure-Mallet M, Jolivet-Gougeon A. Emergence of resistance to antibacterial agents: the role of quaternary ammonium compounds- a critical review. Antimicrobial Agents. 2002;39(5):381–9. 10.1016/j.ijantimicag.2012.01.011 22421329

[21] Tezel U, Pavlostathis SG. Quaternary ammonium disinfectants: microbial adaptation, degradation and ecology. Curr Opin Biotechnol. 2015;33:296–304. doi: 10.1016/j.copbio.2015.03.018 25864173 10.1016/j.copbio.2015.03.018

[22] Cutler RA, Drobeck HP, editors. Toxicology of Cationic Surfactants. New York: Marcel Dekker, Inc.; 1970.

[23] Gosselin RE, Smith RP, Hodge HC. Clinical Toxicology Of Commercial Products. 5 ed. Balitmore: Williams and Wilkins; 1984.

[24] Warren MR, TBecker TJ, Marsh DG, Shelton RS. Pharmacological and Tocicological studies on cetylpiridinium chloride, a new germicide. Journal of Pharmacology and experimental therepeutics. 1942;74(4):401–8.

[25] Zhou X, Handie A, Salari H, Fifer EK, Breen PJ, Compadre CM. High-performance liquid chromatography determination of residue levels on chicken carcasses treated with cetylpyridinium chloride. J Chromatogr B Biomed Sci Appl. 1999;728(2):273–7. 10406212 10.1016/s0378-4347(99)00100-0

[26] Rodriguez-Morales S, Zhou X, Salari H, Castillo R, Breen PJ, Compadre CM. Liquid chromatography determination of residue levels on apples treated with cetylpyridinium chloride. J Chromatogr A. 2005;1062(2):285–9. 15679166 10.1016/j.chroma.2004.11.039

[27] Golbraikh A, Tropsha A. Beware of q(2)! J Mol Graph Model. 2002;20(4):269–76. 11858635 10.1016/s1093-3263(01)00123-1

[28] Gramatica P. Principles of QSAR models validation: internal and external. Qsar Comb Sci. 2007;26(5):694–701.

[29] Martin TM, Harten P, Young DM, Muratov EN, Golbraikh A, Zhu H, et al Does Rational Selection of Training and Test Sets Improve the Outcome of QSAR Modeling? J Chem Inf Model. 2012;52(10):2570–8. doi: 10.1021/ci300338w 23030316 10.1021/ci300338w

[30] Sushko I, Novotarskyi S, Körner R, Pandey AK, Rupp M, Teetz W, et al Online chemical modeling environment (OCHEM): web platform for data storage, model development and publishing of chemical information. Journal of Computer-Aided Molecular Design. 2011;25(6):533–54. doi: 10.1007/s10822-011-9440-2 21660515 10.1007/s10822-011-9440-2 PMC3131510

[31] Tropsha A, Gramatica P, Gombar VK. The importance of being earnest: Validation is the absolute essential for successful application and interpretation of QSPR models. Qsar Comb Sci. 2003;22(1):69–77.

[32] Silverman K. The organic chemistry of drug design and drug action. 2 ed: Elsiver; 2004.

[33] Rath EC, Bai Y. Quantitative Structure-Activity Relation Study of Quaternary Ammonium Compounds in Pathogen Control: Computational Methods for the Discovery of Food Antimicrobials. Journal of Chemical Informatics. 2016;2(1):3–10.

[34] Mohamad IB, Usman D. Standardization and Its Effects on K-Means Clustering Algorithm. Research Journal fo Applied Sciences, Engineering and Technology. 2013;6(17):3299–303.

[35] Gramatica P, Cassani S, Chirico N. QSARINS-chem: Insubria datasets and new QSAR/QSPR models for environmental pollutants in QSARINS. J Comput Chem. 2014;35(13):1036–44. doi: 10.1002/jcc.23576 24599647 10.1002/jcc.23576

[36] Gramatica P, Chirico N, Papa E, Cassani S, Kovarich S. QSARINS: A new software for the development, analysis, and validation of QSAR MLR models. Journal of Computational Chemistry. 2013;34(24):2121–32.

[37] Eriksson L, Jaworska J, Worth AP, Cronin MTD, McDowell RM, Gramatica P. Methods for reliability and uncertainty assessment and for applicability evaluations of classification- and regression-based QSARs. Environ Health Persp. 2003;111(10):1361–75. 10.1289/ehp.5758 PMC1241620 12896860

[38] Hattori M, Okuno Y, Goto S, Kanehisa M. Development of a chemical structure comparison method for integrated analysis of chemical and genomic information in the metabolic pathways. J Am Chem Soc. 2003;125(39):11853–65. doi: 10.1021/ja036030u 14505407 10.1021/ja036030u

[39] Hattori M, Okuno Y, Goto S, Kanehisa M. Heuristics for chemical compound matching. Genome Inform. 2003;14:144–53. 15706529

[40] Hattori M, Tanaka N, Kanehisa M, Goto S. SIMCOMP/SUBCOMP: chemical structure search servers for network analyses. Nucleic acids research. 2010;38(Web Server issue):W652–6. doi: 10.1093/nar/gkq367 20460463 10.1093/nar/gkq367 PMC2896122

[41] Smith TJ. Molview—a Program for Analyzing and Displaying Atomic Structures on the Macintosh Personal-Computer. J Mol Graphics. 1995;13(2):122–5. 10.1016/0263-7855(94)00019-o 7619787

[42] Fazlara A, Ekhtelat M. The Disinfectant Effects of Benzalkonium Chloride on Some Important Foodborne Pathogens. American_Eurasian Journal fo Argicultural and Environmental Science. 2012;12(1):23–9.

[43] Bodor N, Kaminski JJ, Selk S. Soft drugs. 1. Labile quaternary ammonium salts as soft antimicrobials. J Med Chem. 1980;23(5):469–74. 7381846 10.1021/jm00179a001

[44] Mendez-Vilas A. Multidisciplinary Approaches for Studying and Combating Microbial Pathogens: BrownWalker Press; 2015. 151 p.

[45] Sharma S, Gangal S, Rauf A. Convenient one-pot synthesis of novel 2-substituted benzimidazoles, tetrahydrobenzimidazoles and imidazoles and evaluation of their in vitro antibacterial and antifungal activities. Eur J Med Chem. 2009;44(4):1751–7. doi: 10.1016/j.ejmech.2008.03.026 18472189 10.1016/j.ejmech.2008.03.026

[46] Jang KH, Lee Y, Sim CJ, Oh KB, Shin J. Bioactive lipids from the sponge Spirastrella abata. Bioorg Med Chem Lett. 2012;22(2):1078–81. doi: 10.1016/j.bmcl.2011.11.105 22182501 10.1016/j.bmcl.2011.11.105

[47] Ahn M, Murugan RN, Jacob B, Hyun JK, Cheong C, Hwang E, et al Discovery of novel histidine-derived lipo-amino acids: applied in the synthesis of ultra-short antimicrobial peptidomimetics having potent antimicrobial activity, salt resistance and protease stability. Eur J Med Chem. 2013;68:10–8. doi: 10.1016/j.ejmech.2013.07.008 23933046 10.1016/j.ejmech.2013.07.008

[48] Kim HS, Kwon KC, Kim KS, Lee CH. Synthesis and antimicrobial activity of new 3 alpha-hydroxy-23,24-bisnorcholane polyamine carbamates. Bioorg Med Chem Lett. 2001;11(23):3065–8. 11714611 10.1016/s0960-894x(01)00632-1

[49] Martínez-Suárez JV, Ortiz S, López-Alonso V. Potential Impact of the Resistance to Quaternary Ammonium Disinfectants on the Persistence of Listeria monocytogenes in Food Processing Environments. Frontiers in Microbiology. 2016;7:638 doi: 10.3389/fmicb.2016.00638 27199964 10.3389/fmicb.2016.00638 PMC4852299

[50] Mereghetti L, Quentin R, Marquet-Van Der Mee N, Audurier A. Low sensitivity of Listeria monocytogenes to quaternary ammonium compounds. Appl Environ Microbiol. 2000;66(11):5083–6. 11055967 10.1128/aem.66.11.5083-5086.2000 PMC92423

[51] Romanova NA, Wolffs PF, Brovko LY, Griffiths MW. Role of efflux pumps in adaptation and resistance of Listeria monocytogenes to benzalkonium chloride. Appl Environ Microbiol. 2006;72(5):3498–503. doi: 10.1128/AEM.72.5.3498-3503.2006 16672496 10.1128/AEM.72.5.3498-3503.2006 PMC1472371

[52] Brown SD, Traczewski MM. Comparative in vitro antimicrobial activities of torezolid (TR-700), the active moiety of a new oxazolidinone, torezolid phosphate (TR-701), determination of tentative disk diffusion interpretive criteria, and quality control ranges. Antimicrob Agents Chemother. 2010;54(5):2063–9. doi: 10.1128/AAC.01569-09 20231392 10.1128/AAC.01569-09 PMC2863606

[53] Bowker KE, Caspers P, Gaucher B, MacGowan AP. In vitro activities of three new dihydrofolate reductase inhibitors against clinical isolates of gram-positive bacteria. Antimicrob Agents Chemother. 2009;53(11):4949–52. doi: 10.1128/AAC.00845-09 19738027 10.1128/AAC.00845-09 PMC2772311

[54] Lemaire S, Kosowska-Shick K, Appelbaum PC, Verween G, Tulkens PM, Van Bambeke F. Cellular pharmacodynamics of the novel biaryloxazolidinone radezolid: studies with infected phagocytic and nonphagocytic cells, using Staphylococcus aureus, Staphylococcus epidermidis, Listeria monocytogenes, and Legionella pneumophila. Antimicrob Agents Chemother. 2010;54(6):2549–59. doi: 10.1128/AAC.01724-09 20385852 10.1128/AAC.01724-09 PMC2876393

[55] Mohamed MS, Kamel MM, Kassem EM, Abotaleb N, Abd El-Moez SI, Ahmed MF. Novel 6,8-dibromo-4(3H)quinazolinone derivatives of anti-bacterial and anti-fungal activities. Eur J Med Chem. 2010;45(8):3311–9. doi: 10.1016/j.ejmech.2010.04.014 20452707 10.1016/j.ejmech.2010.04.014

[56] Jung ME, Yang EC, Vu BT, Kiankarimi M, Spyrou E, Kaunitz J. Glycosylation of fluoroquinolones through direct and oxygenated polymethylene linkages as a sugar-mediated active transport system for antimicrobials. J Med Chem. 1999;42(19):3899–909. 10508438 10.1021/jm990015b

[57] Haas W, Pillar CM, Zurenko GE, Lee JC, Brunner LS, Morris TW. Besifloxacin, a novel fluoroquinolone, has broad-spectrum in vitro activity against aerobic and anaerobic bacteria. Antimicrob Agents Chemother. 2009;53(8):3552–60. doi: 10.1128/AAC.00418-09 19506065 10.1128/AAC.00418-09 PMC2715578

