# Correction: Identification of potential antimicrobials against *Salmonella typhimurium* and *Listeria monocytogenes* using Quantitative Structure-Activity Relation modeling

**DOI:** 10.1371/journal.pone.0193628

**Published:** 2018-02-23

**Authors:** The PLOS ONE Staff

Tables 2 and 3 incorrectly show only the top 5 compounds against S. typhimurium and L. monocytogenes, respectively, rather than the top 10. The publisher apologizes for these errors. Please see the corrected Tables 2 and 3 here.

**Table 2:** Top 10 compounds against S. typhimurium in terms of log(MIC).

SMILES Structure of Potential Compounds	log(MIC) (μg/ml)		SMILES Structure of Potential Compounds	log(MIC) (μg/ml)
*S*. *typhimurium* (model 76)				
C[C@@]12CC[C@]3(CC[C@H](C3C1CCC4[C@]2(CCC5[C@@]4(CC[C@@H](C5(C)C)O)C)C)C (= C)C[N+]6 = CC = CC = C6)CO		0.606	C[C@@]12CC[C@]3(CC[C@H]([C@@H]3[C@H]1CC[C@H]4[C@]2(CC[C@@H]5[C@@]4(CC[C@@H](C5(C)C)O)C)C)C (= C)C[N+]6 = CC = C(C = C6)N(C)C)C (= O)O.[Br-]	0.7165
CC1 = CC (= C[N+] (= C1)CC (= C)[C@@H]2CC[C@]3(C2C4CCC5[C@]6(CC[C@@H](C(C6CC[C@]5([C@@]4(CC3)C)C)(C)C)O)C)CO)C		0.6082	C[C@H](CCCC(C)C)[C@H]1CC[C@@H]2[C@@]1(CC[C@H]3[C@H]2CC = C4[C@@]3(CC[C@@H](C4)[N+]5 = CC = CC = C5)C)C.I	0.9018
C[C@@]12CC[C@]3(CC[C@H](C3C1CCC4[C@]2(CCC5[C@@]4(CC[C@@H](C5(C)C)O)C)C)C (= C)C[N+]6 = CC = C(C = C6)N(C)C)CO		0.6207	C[C@H](CCCC(C)C)[C@H]1CCC2[C@@]1(CCC3C2CC = C4[C@@]3(CCC(C4)[N+]5 = CC = CC = C5)C)C	0.9018
C[C@@]12CC[C@]3(CC[C@H](C3C1CCC4[C@]2(CCC5[C@@]4(CC[C@@H](C5(C)C)O)C)C)C (= C)C[N+]6 = CC = CC (= C6)CO)CO		0.6251	CC1 = CC = C(C = C1)S (= O) (= O)[O-].CC1 = CC = [N+](C = C1)C2CCC3(C4CCC5(C(C4CC = C3C2)CCC5C(C)CCCC(C)C)C)C	0.9069
C[C@@]12CC[C@]3(CC[C@H](C3C1CCC4[C@]2(CCC5[C@@]4(CC[C@@H](C5(C)C)O)C)C)C (= C)C[N+]6 = CC = C(C = C6)CO)CO		0.6273	CC1 = CC = C(C = C1)S (= O) (= O)O.CC(C)CCCC(C)C1CCC2C1(CCC3C2CC = C4C3(CCC(C4)[N+]5 = CC = CC (= C5)C#N)C)C	0.9268

**Table 3:** Top 10 against L. monocytogenes in terms of log(MIC).

SMILES Structure of Potential Compounds	log(MIC) (μg/ml)	SMILES Structure of Potential Compounds	log(MIC) (μg/ml)
*L*. *monocytogenes* (model 90)			
CCCCCCCCCCCCCCCC[N+]1 = CC = C(C2 = CC = CC = C21)CC(C3 = C(NC4 = CC = CC = C43)C)O	-1.8558	CCCCCCCCCCCCCCCC[N+]1 = CC = C(C = C1)CC	-1.6392
CC1 = CC (= C[N+] (= C1)CC (= O)C2 = CC3 = C(C = C2)C4 = CC = CC = C4C3)C.[Br-]	-1.7315	CCCCCCCCCCCCCCCC[N+]1 = CC = C(C = C1)CCS (= O) (= O)O	-1.6392
CC1 = CC = CC2 = NC (= C(N12)NC(C)(C)CC(C)(C)C)C3 = CC4 = CC = CC = C4C5 = CC = CC = C53	-1.7252	CC(C)(C)CC(C)(C)NC1 = C(N = C2N1C = C(C = C2)Br)C3 = C4C = CC = CC4 = CC5 = CC = CC = C53	-1.6212
CC1 = C[N+] (= CC = C1)CCCCCC2 = CC (= CC = C2)CCCCC[N+]3 = CC = CC (= C3)C	-1.7101	CC(C)(C)CC(C)(C)NC1 = C(N = C2N1C = CC = C2)C3 = C4C = CC = CC4 = CC5 = CC = CC = C53	-1.6212
CC1 = C[N+] (= CC = C1)CCCCCC2 = CC (= CC = C2)CCCCC[N+]3 = CC = CC (= C3)C.[Br-].[Br-]	-1.7101	CC(C)(C)CC(C)(C)NC1 = C(N = C2N1C = CC = C2)C3 = CC4 = CC = CC = C4C5 = CC = CC = C53	-1.6212
