# Supplementary material for: Identification of potential antimicrobials against Salmonella typhimurium and Listeria monocytogenes using Quantitative Structure-Activity Relation modeling
Source: PLoS One. 2017 Dec 13;12(12):e0189580. doi: 10.1371/journal.pone.0189580 (PMC5728541; doi:10.1371/journal.pone.0189580)
Supplement: S1 Table — (DOCX) [file pone.0189580.s001.docx]

**S1. The actual structures of similar compounds for three bacteria species**

| Compound Number | Structure | Compound Number | Structure |
| --- | --- | --- | --- |
| L35 | 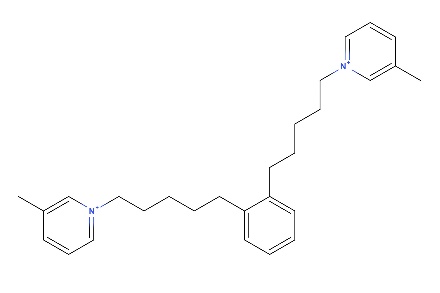 | E38 | 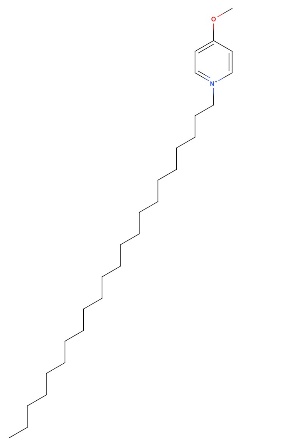 |
| E23 | 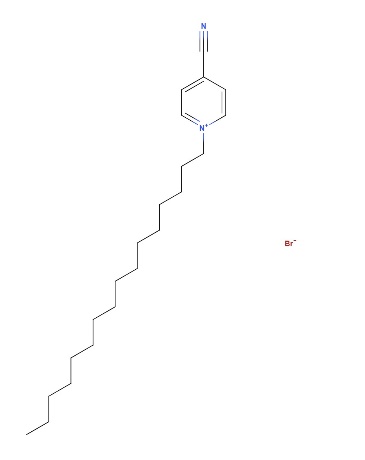 | E39 | 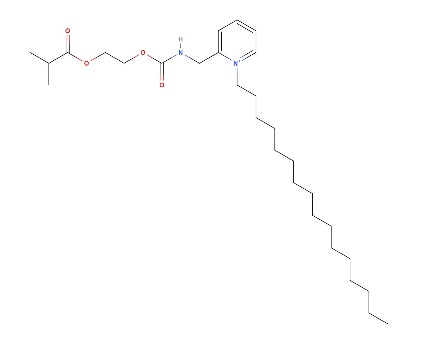 |
| E24 | 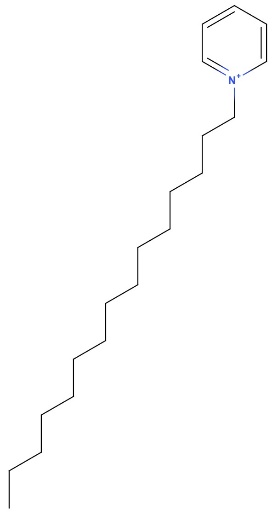 | E40 | 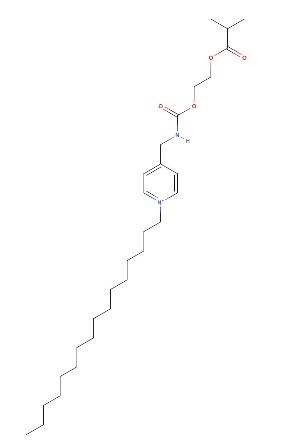 |
| E35 | 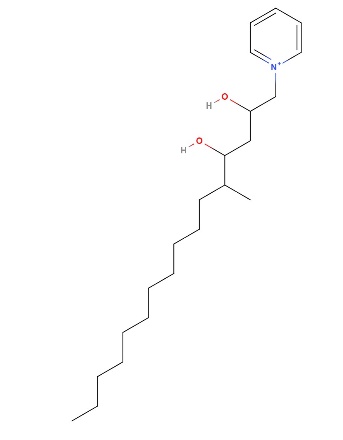 | S16 | 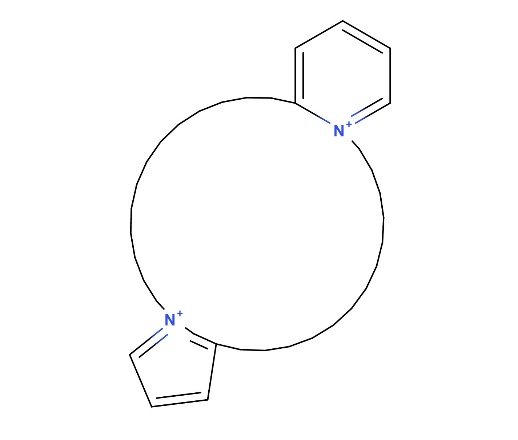 |
| E36 | 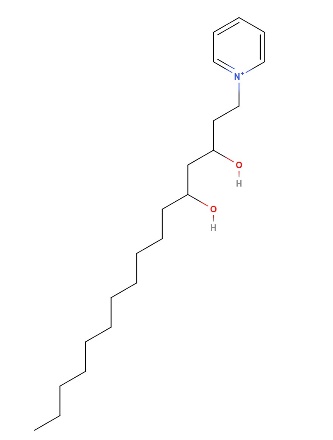 | S36 | 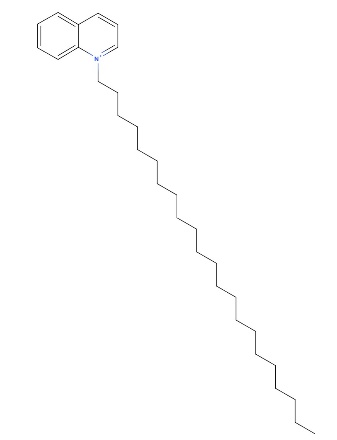 |
| E37 | 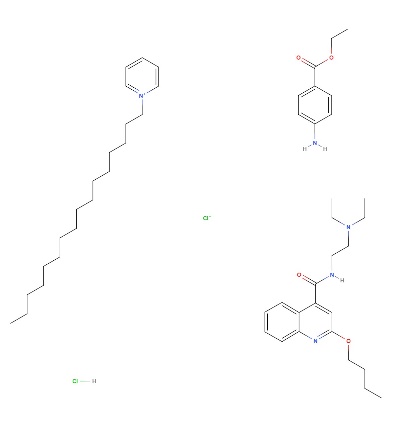 | S37 | 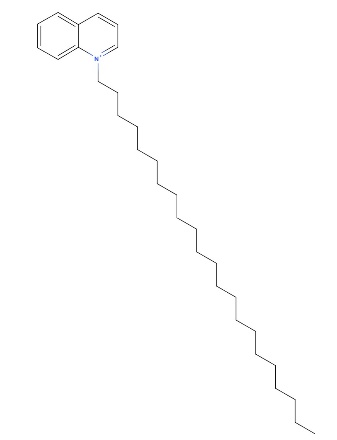 |
|  |  | S39 | 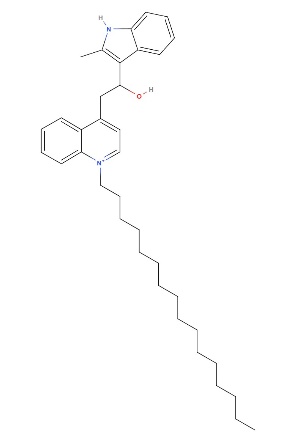 |
